# Healthcare for servicewomen on military missions

**DOI:** 10.1136/jramc-2018-001106

**Published:** 2019-05-24

**Authors:** Jia-Yu Guo, Hui-Ru Hou, F Cao

**Affiliations:** 1 Department of Nephrology, The Second Medical Centre, Chinese PLA General Hospital, Beijing, China; 2 National Clinical Research Centre of Geriatrics Disease, Chinese PLA General Hospital, Beijing, China; 3 Department of Nursing, The Second Medical Centre, Chinese PLA General Hospital, Beijing, China; 4 The Second Medical Centre, Chinese PLA General Hospital, Beijing, China

**Keywords:** servicewoman, healthcare, military mission

## Abstract

Increases in the number of women in critical positions on military missions place new demands for specialised healthcare services to promote performance. The main health problems servicewomen facing are musculoskeletal injuries, reproductive diseases, iron deficiency and mental health problems. Herein, we propose several suggestions based on the rich experiences of our hospital. First is to offer preventive measures for servicewomen health. Second is to equip servicewomen with portable medicine packet to treat common diseases. Third is to provide people-centred integrated care.

## Introduction

The emerging technology-intensive warfare has less dependency on physical strength of military personnel but requires more skills, leading to the increased demand of women into military service. Servicewomen have made great contributions demonstrated by several recent regional wars such as conflicts in Iraq and Afghanistan.^1^ Nowadays, servicewomen have occupied multiple positions and taken great responsibilities beyond their previous roles behind the ‘*front line*’ of the battlefield. They may act as pilot, sailor, special force, astronaut and so on.

Presently, the number of servicewomen in China is increasing. Taking Chinese Navy as an example, women accounted for 5% of the service personnel on China’s first aircraft carrier according to a report in 2012.^2^ They act as nurses, signalman, helmsman and so on. In comparison, previous data reported that women accounted for 10.5% of the service personnel on aircraft carriers of US Navy by the end of 1995.^3^

Given the rising roles of servicewomen, it becomes essential to investigate how to provide them with specific healthcare since women have different characteristics both physically and mentally compared with men, which make them more vulnerable to challenges including extreme pressure of modern warfare, the complexity of operations, harsh battlefield environment and so on. Since 1950s, our hospital has provided high-quality healthcare for servicewomen both in the hospital and during military tasks. Herein, we summarised the characteristics and health problems of servicewomen and raised several proposals for servicewomen healthcare based on the experiences of our hospital.

## Characteristics of servicewomen

Servicewomen possess weaker physical strength and endurance, less force-generating capacity, and higher fatigability than male members, but they demonstrate greater flexibility and better balance.^4^ Moreover, although servicewomen show better emotional stability than servicemen,^5^ they tend to suffer serious mental health problems during missions and conflicts, such as anxiety, depression and even post-traumatic stress disorder.^6 7^ Servicewomen have advantages compared with men during deployment; however, the emerging physical and mental disadvantages need to be focused on, which may increase the risks of health problems and thus poor performance.

## Health problems of servicewomen

The main health problems of servicewomen include injuries, reproductive diseases, iron deficiency and mental health problems.^6 8–11^

### Injury

Servicewomen have a higher probability of suffering from injuries, including training and combat injuries.^8 9^ For training injury, recent research reported that the incidence rate of training injury among servicewomen is about 47% compared with the incidence rate of 27%~32.8% among servicemen.^8^ Besides, servicewomen exhibit a higher proportion of overuse injuries especially in the lower extremity. They are also more likely to get multiple injuries.^12^ For combat injury, a US study reported that explosion, fall, motor vehicle collision, blunt object and machinery were listed as the top 5 causes of injuries among US servicewomen during the conflicts in Iraq and Afghanistan.^9^ Although the incidence of battle-related and explosion-related injuries is lower in servicewomen compared with men, servicewomen sustain similar injury severity and even higher fatality rate than servicemen.

### Reproductive disease

Servicewomen are more vulnerable to reproductive diseases such as genital infection and menoxenia.^10 13^ Chinese researchers investigated the incidence of common reproductive diseases in servicewomen of The Chinese People‘s liberation Army (PLA) from 2008 to 2013. These diseases included inflammatory diseases, menoxenia, infertility, benign tumour and malignant neoplasm. Among them, inflammatory diseases accounted for 60% of reproductive diseases in PLA servicewomen.^10^ Besides, another report indicated that the incidence of female genital bacterial infection (including candidiasis, trichomoniasis, gardnerella) of servicewomen on mission was much higher than that of servicewomen without task in a PLA international rescue team.^14^ Another common disease is menoxenia. The incidence of menoxenia is 16.6% for servicewomen according to a survey by Chinese military hospitals.^10^ For servicewomen on mission, the incidence of menoxenia increases. For example, the incidence of menoxenia is 59.72% in servicewomen on the peacekeeping mission in Congo, and reaches nearly 100% in servicewomen performing tasks in the plateau area.^13 15^

### Iron deficiency

Research showed that body iron levels declined during physical activities, particularly in women.^16^ Iron deficiency may reduce both physical work capacity and cognitive performance.^17^ Chinese researchers conducted a survey in 608 Chinese servicewomen and found that the iron deficiency rate was about 28%.^11^ They also indicated that intense military tasks may increase the risk of iron deficiency in servicewomen.

### Mental health problems

In military missions, mental problems are induced by many factors, such as the threat of war, harsh environment, duty assignments, personal illnesses, loss of support systems and separation from family. A previous Chinese survey reported that 15.8% of servicewomen and 18.2% of servicemen have various mental problems in over 10 000 PLA service members.^5^ These mental problems include obsession, depression, hostility, paranoid ideation and so on. Another research suggested that female navy soldiers who were under 24 years old and had low education level are more likely to be at high risk of psychological problems such as anxiety, depression and sleep disorder.^6^ The incidence of post-traumatic stress disorder is also higher for servicewomen, having experienced conflicts and witnessed death, compared with servicemen.^7^ The outcome of mental problems in women differs from men in military personnel. One research demonstrated that servicewomen were more than twice as likely as servicemen to attempt suicide.^18^

## Proposals to promote servicewomen healthcare

**Figure 1:**
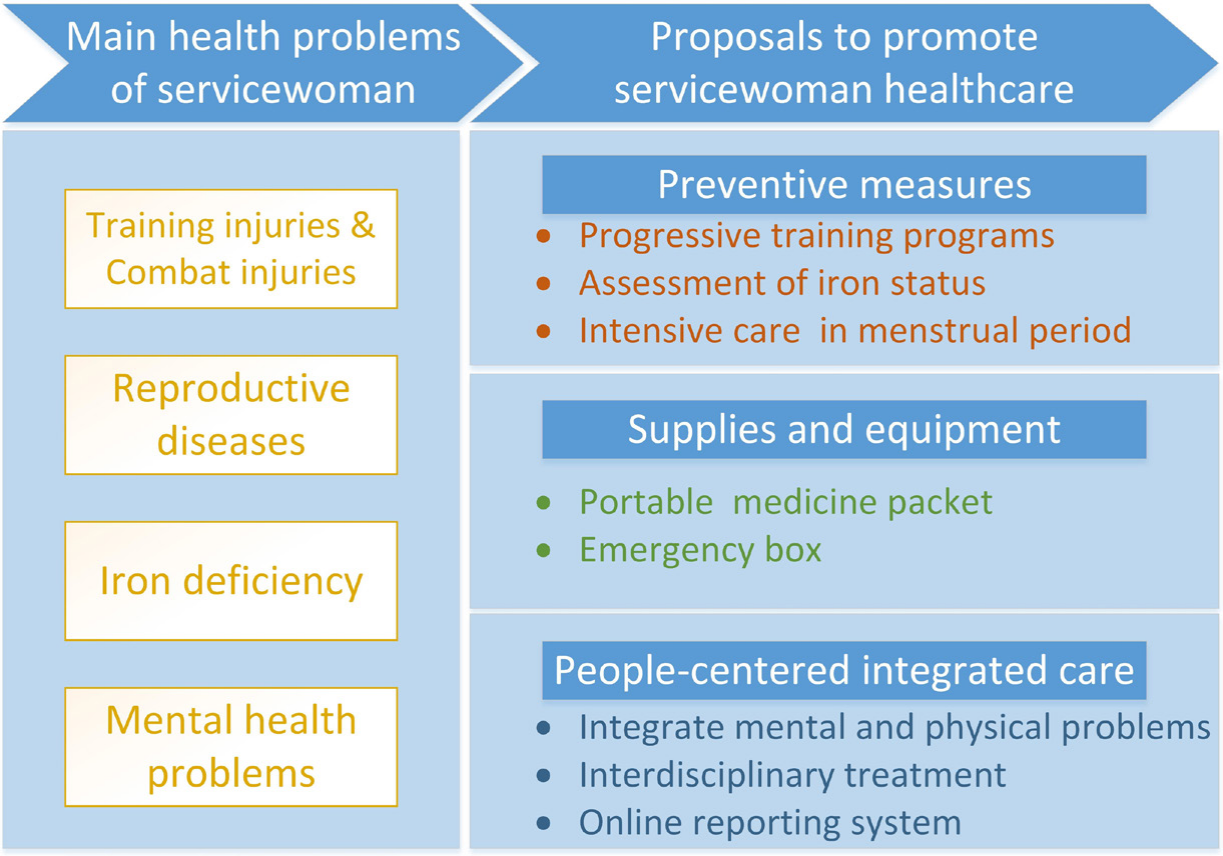
Main health problems of servicewomen and proposals. The figure shows a frame of the core content of the manuscript. The left part demonstrates the main health problems of servicewomen: musculoskeletal injuries, reproductive diseases, iron deficiency and mental health problems. The right part demonstrates the suggestions we propose to address these problems: preventive measures, supplies and equipment to treat common diseases, and people-centred integrated care.

To overcome the health problems of servicewomen, we proposed several suggestions based on the experiences of our hospital (Figure 1).

### Preventive measures for servicewomen health

Identification of gender-specific differences in injury patterns and characteristics would facilitate adjustments in preparation to decrease the risk of injury. For training injuries, daily physical training intensity and testing standards should be formulated on the basis of gender characteristics. Besides, training programmes should be conducted progressively in order to prevent female soldiers from unnecessary injuries. For iron deficiency, it is necessary to assess iron status before combat training. For the indicative signs of reproductive diseases, medical providers should emphasise the importance of reproductive hygiene to all servicewomen and give intensive care to women on menstrual period.

### Portable medicine packet to treat common diseases

To ensure the timely medical supply when performing military tasks, portable medicine packet for every servicewoman is recommended. The packet contains vaginal anti-infection drugs or drugs for other period problems, which meets the basic needs of servicewomen. In addition, we developed various types of emergency boxes possessed by the medical team. The boxes include drug box, nursing box, injection box and so on.

### People-centred integrated care

In order to meet the multiple medical needs of individuals, an interdisciplinary treatment group should be created, which includes a surgeon, a physician, a dietitian, a psychological consultant, a pharmacist, several nurses and so on. Medical providers should not only treat a single disease but also take all kinds of problems into consideration. For example, although the mental health status of servicewomen is better than that of servicemen during peacetime,^5^ servicewomen on military missions suffered from mental problems more frequently induced by conflicts and injuries than servicemen.^7^ The incidence rate of menoxenia increased considerably when performing tasks compared with in daily time.^10 13^ The complex health issues urge medical providers to integrate mental and physical health problems to address the multiple healthcare needs of servicewomen. To help achieve this goal, our hospital has built an online reporting system to record and assess the overall health status of servicewomen.

## Summary

Servicewomen have exhibited increasing roles and made great contributions to national defence. Therefore, all medical providers should be fully aware of the importance of healthcare for servicewomen. Given the special physical and mental status of women, specific treatments should be provided in order to promote their health. The Chinese military force has made big progress in providing effective healthcare and medical treatment for servicewomen. In the future, we as medical providers will continue our work on promoting health benefits for servicewomen, which in turn will promote military ability.

## References

[1] Jones S Perspective. US Army Med Dep J 2014:1–2. 25864230

[2] Ning SE, Wang WD, Wei G Impact of female soldiers deployed aboard naval vessels on seamen’s psychology in China. J Prev Med Chin PLA 2017;35:1444–58.

[3] Fitzgerald AS, Duboyce RL, Ritter JB, et al A primer on the unique challenges of female soldiers' reproductive issues in a war-ready culture. Military Medicine 2013;178:511–6. 10.7205/MILMED-D-12-00384 23756009

[4] Allison KF, Keenan KA, Sell TC, et al Musculoskeletal, biomechanical, and physiological gender differences in the US military. US Army Med Dep J 2015:22–32. 26101903

[5] Liu JL, Liu YB, Feng ZZ, et al A study on the character of the mental health and their related factor of military personnel. Chinese Journal of Health Psychology 2005;13:423–7.

[6] Xia SY, Chen L, HB W The survey of the mental health of female soldiers during the mission of ‘harmony mission-2013’. People’s Military Surgeon 2014;57:949–50.

[7] Olff M, Langeland W, Draijer N, et al Gender differences in posttraumatic stress disorder. Psychological Bulletin 2007;133:183–204. 10.1037/0033-2909.133.2.183 17338596

[8] Xu Y, Sun JH, Wang XW, et al Analysis and measures of risk factors of military training injuries among female soldiers.. People’s Military Surgeon 2016;59:122–4.

[9] Hylden C, Johnson AE, Rivera JC Comparison of female and male casualty cohorts from conflicts in Iraq and Afghanistan. US Army Med Dep J 2015:80–5. 26101910

[10] Yin GP, AF W, Qi L, et al General investigation of gynecologic diseases of PLA Servicewomen:2008 to 2013. Med &amp; Pharm J Chin PLA 2014;26:96–9.

[11] Wang YL, Zhu RM, HL Y, et al Prevalence of iron deficiency and health-related quality of life among female military personnel.. Journal of Medical Forum 2010;31:40–2.

[12] Sauers SE, Scofield DE Strength and conditioning strategies for females in the military. Strength and Conditioning Journal 2014;36:1–7. 10.1519/SSC.0000000000000060

[13] JH L, Wang HL, Xue Y, et al Analysis on menstrual disorders and influencing factors among female soldiers in peace-keeping forces.. J Prev Med Chin PLA 2014;32:336–7.

[14] Zhu TY, Yin GP, Chen M, et al An epidemiological investigation of the incidence of reproductive tract inflammation of female military staff in an international medical rescue team of one military area command during a rescue mission: prevention and treatment strategies. Med &amp; Pharm J Chin PLA 2014;26:101–3.

[15] Yang XY, Chen HH, Wang CY, et al The influence of the plateau environment on the menstruation of female soldiers in the mission of rushing into the plateau.. Med J Nat Defending Forces in North China 2010;22:479–80.

[16] McClung JP, Martini S, Murphy NE, et al Effects of a 7-day military training exercise on inflammatory biomarkers, serum hepcidin, and iron status. Nutr J 2013;12 10.1186/1475-2891-12-141 PMC3830559 24188143

[17] McClung JP, Murray-Kolb LE Iron nutrition and premenopausal women: effects of poor iron status on physical and neuropsychological performance. Annu. Rev. Nutr. 2013;33:271–88. 10.1146/annurev-nutr-071812-161205 23642204

[18] Wise J Army suicide attempts are most likely among enlisted soldiers on first tour of duty and female soldiers, US Study finds. BMJ 2015;8 10.1136/bmj.h3702 26160382

